# Unravelling the cellular and molecular pathogenesis of bovine babesiosis: is the sky the limit?

**DOI:** 10.1016/j.ijpara.2018.11.002

**Published:** 2019-02

**Authors:** Carlos E. Suarez, Heba F. Alzan, Marta G. Silva, Vignesh Rathinasamy, William A. Poole, Brian M. Cooke

**Affiliations:** a Department of Veterinary Microbiology and Pathology, Washington State University, Pullman, WA, United States; b Animal Disease Research Unit, Agricultural Research Service, USDA, WSU, Pullman, WA, United States; c Parasitology and Animal Diseases Department, National Research Center, Dokki, Giza, Egypt; d Department of Microbiology, Biomedicine Discovery Institute, Monash University, Victoria 3800, Australia

**Keywords:** *Babesia*, Babesiosis, Tick fever, Parasite vaccines, Pathogenesis

## Abstract

The global impact of bovine babesiosis caused by the tick-borne apicomplexan parasites *Babesia bovis*, *Babesia bigemina* and *Babesia divergens* is vastly underappreciated. These parasites invade and multiply asexually in bovine red blood cells (RBCs), undergo sexual reproduction in their tick vectors (*Rhipicephalus* spp. for *B. bovis* and *B. bigemina*, and *Ixodes ricinus* for *B. divergens*) and have a trans-ovarial mode of transmission. *Babesia* parasites can cause acute and persistent infections to adult naïve cattle that can occur without evident clinical signs, but infections caused by *B. bovis* are associated with more severe disease and increased mortality, and are considered to be the most virulent agent of bovine babesiosis. In addition, babesiosis caused by *B. divergens* has an important zoonotic potential. The disease caused by *B. bovis* and *B. bigemina* can be controlled, at least in part, using therapeutic agents or vaccines comprising live-attenuated parasites, but these methods are limited in terms of their safety, ease of deployability and long-term efficacy, and improved control measures are urgently needed. In addition, expansion of tick habitats due to climate change and other rapidly changing environmental factors complicate efficient control of these parasites. While the ability to cause persistent infections facilitates transmission and persistence of the parasite in endemic regions, it also highlights their capacity to evade the host immune responses. Currently, the mechanisms of immune responses used by infected bovines to survive acute and chronic infections remain poorly understood, warranting further research. Similarly, molecular details on the processes leading to sexual reproduction and the development of tick-stage parasites are lacking, and such tick-specific molecules can be targets for control using alternative transmission blocking vaccines. In this review, we identify and examine key phases in the life-cycle of *Babesia* parasites, including dependence on a tick vector for transmission, sexual reproduction of the parasite in the midgut of the tick, parasite-dependent invasion and egression of bovine RBCs, the role of the spleen in the clearance of infected RBCs (IRBCs), and age-related disease resistance in cattle, as opportunities for developing improved control measures. The availability of integrated novel research approaches including “omics” (such as genomics, transcriptomics, and proteomics), gene modification, cytoadhesion assays, RBC invasion assays and methods for in vitro induction of sexual-stage parasites will accelerate our understanding of parasite vulnerabilities. Further, producing new knowledge on these vulnerabilities, as well as taking full advantage of existing knowledge, by filling important research gaps should result in the development of next-generation vaccines to control acute disease and parasite transmission. Creative and effective use of current and future technical and computational resources are needed, in the face of the numerous challenges imposed by these highly evolved parasites, for improving the control of this disease. Overall, bovine babesiosis is recognised as a global disease that imposes a serious burden on livestock production and human livelihood, but it largely remains a poorly controlled disease in many areas of the world. Recently, important progress has been made in our understanding of the basic biology and host-parasite interactions of *Babesia* parasites, yet a good deal of basic and translational research is still needed to achieve effective control of this important disease and to improve animal and human health.

## Highlights

• Defining vulnerabilities in the life-cycle of *Babesia* parasites may lead to designing new control measures for babesiosis.
• Poorly understood interactions between *Babesia* parasites and their hosts can be effectively addressed with new technologies.
• Effective control of babesiosis will require additional extensive integrative research strategies.

## 1 Introduction

Apicomplexans are obligate intracellular parasites defined by the presence of a cell invasion apparatus consisting of rhoptries, micronemes, and conoid organelles, known as the apical complex, which facilitates invasion of these parasites into their host red blood cells (RBCs). Members of *Babesia*, of which more than 100 species have been described (Chauvin et al., 2009), reside within the order of Piroplasmida and can infect domestic and wild vertebrates as well as becoming an emerging pathogen of humans, of particular importance in transfusion medicine (Lobo et al., 2013). These tick-borne apicomplexan parasites are trans-ovarially transmitted by Ixodidae ticks and similar to other related Alveolata, including dinoflagellates, they contain a remnant membrane-bound, DNA-containing plastid organelle, derived from a vestigial endosymbiotic photosynthetic organism, most likely a red alga (Arisue and Hashimoto, 2015). *Babesia* parasites contain most apicomplexan invading organelles, except conoids. The evolutionary origin of *Babesia* parasites, similar to other apicomplexans, is most probably related to a photosynthetic colpodellid-like organism (Kuvardina et al., 2002, Leander et al., 2003, Mathur et al., 2018). How apicomplexans switched from an autotrophic to a parasitic life-style is currently an active field of research that may provide clues towards our understanding of the mechanisms involved in the evolution of virulence in these organisms of both veterinary and medical importance.

Babesiosis is a globally important tick-borne disease caused by apicomplexan parasites of the genus *Babesia*. *Babesia* spp. are highly successful intracellular parasites, which acquired highly sophisticated mechanisms for survival during long term co-evolution with their hosts. As a result, these parasites have developed a complex life-cycle involving hard ticks as definitive hosts and vertebrates as intermediate hosts. Thus, *Babesia* spp. invade and multiply asexually by binary fission inside RBCs of their infected vertebrate hosts and produce sexual forms in the midgut of the ixodid tick vectors, their definitive hosts, where they undergo sexual multiplication (Mehlhorn and Shein, 1984).

Bovine babesiosis is mainly caused by *Babesia bovis* and *Babesia bigemina* in tropical and sub-tropical regions worldwide, and these parasites remain by far the most studied agents of tick fever. In addition, *Babesia divergens* is responsible for bovine babesiosis in Europe and an important zoonotic pathogen that may also infect immunocompromised humans and can cause a potentially life-threatening disease (Beugnet and Moreau, 2015, Rozej-Bielicka et al., 2015). Bovine babesiosis has an enormous economic and social impact on beef and dairy industries worldwide (Suarez and Noh, 2011). Despite the availability and use of live vaccines comprising attenuated parasites (De Vos and Bock, 2000, Florin-Christensen et al., 2014) in a few countries, bovine babesiosis remains poorly controlled, confirming the urgent need for novel vaccines to prevent the development of acute disease and expansion of the parasites into non-endemic areas. Currently, the control of bovine babesiosis is increasingly under threat due to climatic change that favors vector development and expansion, and other environmental factors (Dantas-Torres, 2015, Sonenshine, 2018). Recent investigations in the biology of the tick-borne apicomplexan *Babesia* parasites revealed an impressive repertoire of resources available to these parasites, allowing them to survive the constant changes and pressures occurring in their environments, including overcoming the hostile activity of the host’s immune system. While *Babesia* parasites are able to use a seemingly unlimited and diverse repertoire of resources to survive and develop in their tick vector and vertebrate hosts, this also suggests increasing challenges in our search for new resources and strategies toward improved control, treatment and preventative measures. However, as our research toolbox expands due to emergence of novel technologies, our knowledge of the complexities in parasite biology and host-parasite interactions also increases at a faster rate.

Improved control of babesiosis relies on practical, sensitive and specific methods for serological and molecular diagnosis of the disease. Diagnostic and genotyping methods are useful not only to demonstrate the presence and impact of the disease in the field, but also to inform on the complexity of the strain variation among and between *Babesia* populations, their relative virulence and the presence of co-infections. In addition, the variable results usually derived from the application of serological diagnostic methods based on apparently widely conserved antigens can provide important clues on the antigenicity of key molecules that can be required to elicit protective immunity. Serological diagnosis can also inform on the evolution of the immune responses in endemic areas, since diagnostic data can provide valuable evidence that may suggest hypotheses on the mechanisms involved in the variations of the antigens involved. Therefore, diagnostic tools are also highly relevant for vaccine development, and for controlling the disease. Control of babesiosis can also be achieved by the elimination of the parasites’ tick vectors using acaricides. So far, none of the strategies applied to control babesiosis have succeeded in complete elimination of the disease in endemic areas, with the sole exception of a long-term campaign (the Cattle Fever Tick Eradication Program) that was developed in the United States (USA) from ∼1906–1943 and beyond, based on the eradication of *Rhipicephalus* ticks (https://www.aphis.usda.gov/animal_health/animal_diseases/tick/downloads/draft_eis_document.pdf). This campaign succeeded under very special circumstances (Bowman, 2006) and could not be reproduced elsewhere, despite important efforts using a similar approach developed in several countries including, for instance, major beef producers such as Australia and Argentina (Pegram et al., 2000, Bowman, 2006). In addition to the development of acaricide resistance in ticks, the impact that would result as a consequence of the application of the massive amounts of acaricides required for a large-scale tick eradication campaign would be unacceptable nowadays under current environmental and public health standards.

In this review, we focus on recounting what is currently known about some of the key mechanisms in the life cycle of *Babesia* parasites and the potential application of novel research strategies to better understand these mechanisms, with the expectation that well-aimed and focused, basic and translational research, will succeed in finding “Achilles heels” that can be exploited to our advantage in the fight against these highly evolved and globally important parasites.

## 2 Current methods of control

Currently, control of bovine babesiosis is mainly based on the use of drugs, live vaccines and tick vector control strategies. In endemic areas, drugs are of limited use since they cannot be used to prevent infections, are expensive, and may leave residual metabolites in milk and/or meat. In addition, successful drug treatment is dependent on early diagnosis, correct administration, and proper storage of the drugs. For bovine babesiosis, and babesiosis in other animals (such as infections caused by *Babesia caballi* for example), imidocarb dipropionate and diminazene aceturate are the usual treatment of choice (Vial and Gorenflot, 2006, Mosqueda et al., 2012). However, continued or inappropriate use of drugs can lead to drug resistance (Mosqueda et al., 2012) and they have the risk of generating chemical residues in meat and milk with potential toxic effects (Coldham et al., 1995), so researchers urgently need to find or develop new alternative effective and affordable drugs with low toxicity. Recent work resulted in the identification of several new drug candidates (Table 1). Studies have shown that strategies based on the combination of chemotherapeutics are significantly more effective for the elimination of parasites, and importantly, result in reduced risk of development of drug-resistance than the application of a single drug therapy (Pritchard et al., 2013). Advantages of a combination of chemotherapeutics include high effectiveness, reduced dosage (which may lead to reduced toxic side effects), lower induction of drug resistance, and recrudescence. To this end, it is also preferable to define drugs that are able to inhibit distinct pathways, leading to synergistic effects, an approach that can also help in preventing development of anti-drug resistance (Lawres et al., 2016). In most apicomplexan parasites a mitochondrial and apicoplast organelle are commonly found. The apicoplast is a plastid like organelle that is described as essential for long-term parasite viability and is therefore considered a good target for specific drugs (Fichera and Roos, 1997, Tuvshintulga et al., 2017). The mitochondria were likely obtained through an endosymbiotic process by incorporation of an α-proteobacterium (Gray et al., 1999, van der Giezen, 2011). Apicomplexan mitochondria present as an interesting target due to distinct differences compared with the mammalian host which has led to the development of anti-parasitic therapeutics, as reviewed in Monzote and Gille (2010). In addition, although chemotherapeutics can be effective in preventing the death of infected animals, they may interfere with the process of developing protective immunity in the herd, and thus are not considered an ideal method of control. The use of live vaccines based on attenuated parasites is the cornerstone to control bovine babesiosis in several countries such as Australia, Israel, and Argentina. However, such live vaccines are also cumbersome and expensive to produce, and have many limitations such as the need of a cold chain, contamination with other infectious agents, a high risk of reversion to virulence due to several possible mechanisms, can only be applied to young calves, etc. Yet, while safer sub-unit vaccines are not available, they are, arguably, the most sustainable and desirable method for control of bovine babesiosis (Brown et al., 2006, Florin-Christensen et al., 2014). *Babesia* strains consist of several subpopulations of parasites that may have variable degrees of virulence. It has been theorised that the occurrence of a virulent or attenuated virulence phenotype of a strain depends of the sum of the contributions of each subpopulation, and hence, virulence was characterised as a “fluid” phenotype (Lau et al., 2011). Live vaccines are usually based on attenuated parasites that are derived from virulent strains by “quick passage” (*B. bovis*), or “slow passage” (*B. bigemina*) in splenectomised (for *B. bovis*) or spleen-intact calves (for *B. bigemina*). The resulting production of pairs of virulent and derived-attenuated strains, generated by this process, suggested the possibility of comparative genomic and transcriptomic studies towards identifying virulence and/or attenuation factors (Lau et al., 2011, Pedroni et al., 2013, Gallego-Lopez et al., 2018).

**Table 1:** Drugs used to inhibit the growth of bovine *Babesia* parasites.

Drug	Molecular target	In field use	*Babesia* spp.	Stage		IC_50_	LD_100_	References
				In vivo	In vitro	(μM)	(μM)	
Diminazene aceturate (DA)	Inhibits the mitochondrial topoisomerase II^a^	Yes	*B. bovis* *B. bigemina* *B. divergens*	Yes (3–5 mg/kg)^b^ Yes (3–5 mg/kg)^b^ Yes (3–5 mg/kg)^b^	Yes Yes Yes	0.19 ± 0.04^c^ 0.48 ± 0.09^d^ 1.85 ± 0.1^c^ 0.21 ± 0.06^d^ 0.38 ± 0.04^c^ 0.14 ± 0.03^d^		^b^Kuttler (1981). ^a^Peregrine and Mamman (1993), ^d^Rizk et al. (2017), ^c^Guswanto et al. (2018)
Imidocarb dipropionate	Interference with the production/use of polyamines^e^, or with the entry of inositol into the parasitised erythrocyte^f^	Yes	*B. bovis* *B. bigemina*	Yes (1–3 mg/kg)^b^ Yes (1–3 mg/kg)^b^	Yes Yes	8.6 nM^g^ 0.08 nM^h^		^e^Bacchi et al. (1981), ^b^Kuttler (1981), ^f^McHardy et al. (1986), ^g^Nott et al. (1990), ^h^Silva et al. (2018b)
Draxxin® (Tulathromycin)	Interference with protein synthesis. 23S prokaryotic rRNA	Yes, but not for babesiosis	*B. bovis* *B. bigemina*	No No	Yes Yes	0.02 ± 0.0006 0.006 ± 0.0002	0.04 0.01	Silva et al. (2018b)
N-acetyl-l-cysteine		No	*B. bovis* *B. bigemina* *B. divergens*	No No No	Yes Yes Yes	332.1 ± 33.1 229.2 ± 37.5 117.2 ± 8.0		Rizk et al., (2017)
Clofazimine	Associated with enhanced activity of phospholipase A2	No	*B. bovis* *B. bigemina*		Yes Yes	4.5 ± 0.30 3.0 ± 0.20		Tuvshintulga et al. (2016)
Nitidine chloride	Topoisomerases	No	*B. bovis* *B. bigemina*	No No	Yes Yes	1.01 ± 0.2 5.34 ± 1.0	4 10	Tayebwa et al. (2018)
Camptothecin	Topoisomerases	No	*B. bovis* *B. bigemina*	No No	Yes Yes	11.67 ± 1.6 4.00 ± 1.0	48 8	Tayebwa et al. (2018)
17-dimethylaminoethylamino-17-demethoxygeldanamycin (17-DMAG)	Heat shock protein 90	No	*B. bovis* *B. bigemina* *B. divergens*	No No No	Yes Yes Yes	0.08 ± 0.0029 0.06 ± 0.002 0.18 ± 0.003		Guswanto et al. (2018)
Atovaquone (AV)	Inhibits the rate of oxygen consumption^i^	Yes, but not for babesiosis	*B. bovis* *B. bigemina* *B. divergens*	No No Yes (1 mg/kg)^j^	Yes Yes Yes	0.03 ± 0.002^c^ 0.71 ± 0.04^c^ 0.01 ± 0.001^c^		^j^Pudney and Gray (1997), ^i^Murphy and Lang-Unnasch (1999), ^c^Guswanto et al. (2018)
DA + AV		No	*B. bovis* *B. bigemina* *B. divergens*	No No No	Yes Yes Yes	0.75 1.06 1.21		Guswanto et al. (2018)
17-DMAG + AV		No	*B. bovis* *B. bigemina* *B. divergens*	No No No	Yes Yes Yes	1.26 1.18 2.07		Guswanto et al. (2018)
17-DMAG + DA		No	*B. bovis* *B. bigemina* *B. divergens*	No No No	Yes Yes Yes	0.87 1.35 1.85		Guswanto et al. (2018)
Trifluralin analogues	Disrupt microtubules	No	*B. bovis* *B. bigemina*	No No	Yes Yes	18.7–8.5^k^ 1.01–8.2^l^		^k^Silva et al. (2013, ^l^^2016^)

*Note:* In vivo dosage is per kg of body weight.

IC_50_, half maximal inhibitory concentration; LD_100_, letal dose, 100%.

## 3 Parasite vulnerabilities

**Fig. 1:**
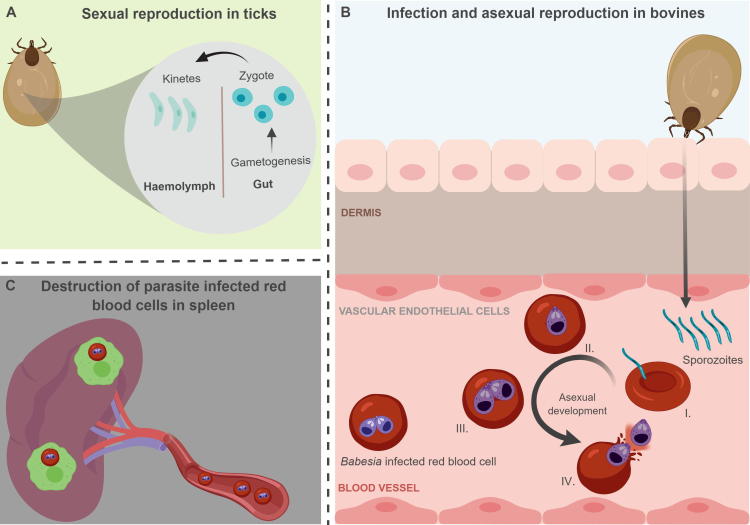
Schematic representation of a simplified and partial life cycle of *Babesia* parasites. (A) Representation of the life-cycle of *Babesia* parasites in an adult, female tick (e.g. *Rhipicephalus microplus*) after taking a blood meal from an infected animal. Upon ingestion, the parasite develops sexual forms that fuse to form zygotes. Zygotes mature into kinetes upon invasion of the midgut epithelial cells of the tick which invade the tick hemolymph, where they can invade the ovaries and ultimately infect the larvae of the next generation of ticks. (B) Representation of *Babesia* infection and asexual reproduction in the bovine host. Sporozoites are introduced from tick saliva into the blood of their bovine host during blood feeding. (I) Sporozoites invade red blood cells (RBCs) and undergo asexual development. (II) Sporozoites mature into trophozoites inside the infected RBC. (III) Trophozoites divide asexually into two daughter merozoites inside infected RBCs. (IV) Merozoites are released into the blood following RBC lysis and then rapidly invade new RBCs. (C) Representation of splenic macrophage-mediated destruction of *Babesia*-infected RBCs in the blood circulation. Figure generated using BioRender.

Despite their similarities, *B. bovis* and *B. bigemina* also have fundamental differences. Both parasites use similar tick vectors including *Rhipicephalus microplus* and *Rhipicephalus annulatus*, although *B. bigemina* has an extended range of potential tick vectors including *Rhipicephalus decoloratus*. In addition, although both parasites are able to transmit transovarially, the larvae are the infective stage for *B. bovis*, whereas nymphs are responsible for transmitting *B. bigemina* during tick feeding, which can be considered as a vulnerability (V#1; Table 2, Fig. 1) that can be used for controlling the parasite). *Babesia bovis* is usually considered more virulent than *B. bigemina*, and tends to cause more dramatic clinical signs and symptoms, such as vascular sequestration and vaso-occlusion and, overall, increased mortality in the vertebrate host. *Babesia divergens* and *Babesia major* are the causative agents of bovine babesiosis in Europe, with *B. divergens* being the more common cause of infection (Zintl et al., 2003, Michelet et al., 2014; Wilson, S.G. (1966). The incidence of *Babesia major* in the Netherlands. In Corradetti, A., (Ed.) Proceedings of the First International Congress of Parasitology, Pergamon, pp. 276–277). *Babesia ovata* and *Babesia orientalis* are predominantly found in Asia with *B. orientalis* being the major causative agent of babesiosis in buffalo (Sivakumar et al., 2016, He et al., 2017). The tick vectors for these *Babesia* parasites have been reviewed elsewhere (Uilenberg, 2006).

**Table 2:** Parasite vulnerabilities and intervention strategies to protect against bovine babesiosis.

Vulnerability #	Justification	Targets – strategies
1: Ticks needed for transmission	Tick control impedes the expansion of the parasite	Tick vaccines, new acaricides, management strategies
2: Sexual reproduction in midgut, invasion of tick tissues	Antibodies against tick-specific stages may interfere with sexual cell fusion in the midgut and other tick stages	Sexual stage-specific *Babesia* antigens: HAP2, 6-Cys, CCp, CPW-WPC
3: RBC invasion	Discovering key molecules involved in the process of parasite attachment and invasion can lead to the development of invasion-interfering strategies	*Babesia* surface exposed antigens: MSAs, RAP-1, CLAMP, MIC-1, TRAP, AMA-1
4: IRBC egression	Interfering with mechanisms for egression can be targeted by drugs	Drugs that inhibit egression such as bumped kinases
5: Trapping of IRBC by the spleen	*Babesia bovis* avoids trapping using sequestration, a mechanism that can be a target for interference	Antigens exposed in the erythrocyte surface, Ves1 and Ves2 antigens, SmORFs? Spherical body proteins?
6: Young calves have increased resistance compared to older	Discovering the bases for increased resistance to bovine babesiosis in calves may guide vaccine design	Immuno-stimulants, interleukins, vaccine adjuvants able to bias the immune responses

RBC, red blood cell; IRBC, infected RBC; HAP2, HAPLESS2/GCS1; MSAs, Merzoite Surface Antigens; Rap-1, Rhoptry Associated Protein-1; MIC-1, Microneme-1; TRAP, thrombospondin-related anonymous protein-1; AMA-1, Apical membrane antigen-1; Ves, Variable erythrocyte surface; SmORF, Small Open Reading Frame.

*Babesia* parasites are able to cause both acute and persistent infections in their vertebrate hosts, where they solely invade RBCs, and depend on female ticks feeding on an infected host for subsequent transovarial transmission (V#2, Table 2, Fig. 1). *Babesia* parasites have a complex life-cycle in their tick definitive host, as briefly described here, and expanded later in the review (Fig. 1). When a tick acquires parasites during blood feeding on a *Babesia*-infected animal, the parasites undergo sexual reproduction in the tick midgut, leading to the production of kinetes that invade the ovaries resulting in eggs in the next generation of ticks being infected (transovarial transmission). Further, sporozoites that reside and reproduce asexually in the salivary glands of the larvae of the next generation of ticks (Mehlhorn and Shein, 1984) are transmitted to a new bovine host during the blood feeding activity of larval (for *B. bovis*) or nymphal (for *B. bigemina*) stages, thereby completing the lifecycle. The impact of *Babesia* spp. infections on the tick has not been thoroughly characterised, but some evidence suggests that infection may result in an overall decrease in tick fitness (de Vos et al., 1989, Cen-Aguilar et al., 1998, Florin-Christensen and Schnittger, 2009).

### 3.1 Transmission of *Babesia* parasites

Control at the level of transmission of *Babesia* parasites may be achieved using a combination of strategies including measures targeting the tick vectors and sexual stages of the parasites. Novel approaches include the development of anti-tick vaccines as an alternative to acaricides, which can generate resistance and may have negative impacts on the environment. The development of tick vaccines was recently reviewed by Valle and Guerrero (2018).

Understanding the mechanisms involved in sexual reproduction of the parasites and their transmission by ticks is also essential for development of novel control strategies aimed at impairing the expansion of the parasite in endemic areas. This can be achieved, at least in theory, by the development of effective transmission-blocking vaccines (TBVs) which is an important goal for controlling arthropod-transmitted parasites in general. It is currently accepted that gametogenesis in *Babesia* spp. occurs mainly in the gut of the tick. As a result of fertilisation of macrogametes by the microgametes, oocytes emerge which then invade epithelial cells of the tick midgut. Kinetes then emerge from infected gut cells into the circulation in the tick haemolymph. These forms are able to migrate to the ovary of the ticks, as well as other tick tissues, where they penetrate into the eggs of the tick progeny, resulting in transovarial transmission. The process culminates with the invasion of the cells in the salivary gland of tick larvae, where sporozoites are formed. The sporozoites are the infective stage which is injected into the blood and s.c. tissue of the next bovine host upon feeding by larval (*B. bovis*) or nymphal (*B. bigemina*) tick stages (Suarez and Noh, 2011). The development of TBVs relies heavily on the discovery of parasite antigens that are differentially expressed in sexual stages of *Babesia* parasites, and testing of the effect of antibodies directed against such key antigens while parasites reside in the midgut of tick vector. However, other approaches targeting other tick stages of the parasites such as kinetes are also at least theoretically possible.

Recent work has identified several genes and gene families of *B. bovis* that are differentially expressed in tick stages. The first of such studies focused on the members of the CCp and *6-cys* gene families. A highly conserved family of six multi-domain adhesion proteins, termed the CCp family, was initially identified in *Plasmodium* parasites. Importantly, it was demonstrated that the CCp proteins in *Plasmodium* are expressed on the surface of gametocytes, and that knock-out (KO) of the *Plasmodium* CCp genes blocks sporozoite formation, thus preventing the development of the parasite in the mosquito vectors (Pradel et al., 2004, Lavazec et al., 2009). A feature of the CCp proteins is the presence of at least one signature domain, Limulus coagulation factor C (LCCL), and additional adhesion domains, suggesting that these proteins may mediate cell contact of gametocytes (Delrieu et al., 2002, Pradel et al., 2004, Simon et al., 2009). These data suggest that CCp proteins are potential targets for the development of TBVs to control the expansion of *Plasmodium* parasites (Pradel et al., 2004). Furthermore, at least three CCp genes (*ccp1*, *ccp2* and *ccp3*) were also identified in *B. bovis* and *B. bigemina*. Experiments performed in an in vitro system for induction of sexual stage forms of *B. bigemina* demonstrated significant upregulation of the expression of all three CCp genes upon induction. Increased production of all three CCp proteins was also found in induced sexual forms of *B. bigemina* using immunoblotting and immunofluorescence (Bastos et al., 2013). In contrast, no expression of the CCp genes could be detected in samples collected from ticks infected with *B. bovis*, and further work will be required in order to clarify the pattern of expression of the CCp proteins in this parasite (Bastos et al., 2013).

Work in *Plasmodium* has also resulted in the initial identification of the 6-Cys gene family. Three of the *Plasmodium* 6-Cys genes (Pfs230, Pf48/45 and Pfs47) are expressed in gametes and are prime candidates for the development of TBVs against *Plasmodium* (Williamson, 2003, Eksi et al., 2006). Main features of 6-Cys proteins include the presence of a domain defined by a conserved arrangement of 6-cysteine residues, and other alternative arrangements (Silva et al., 2011, Alzan et al., 2016b). In silico comparative genomic analysis revealed the presence of at least 10 6-Cys domain-containing protein-coding orthologous genes in *B. bovis* (Silva et al., 2011, Alzan et al., 2016b). Transcription and immunoblot analyses demonstrated that at least two members of the *B. bovis* 6-Cys gene family, denominated 6-Cys A and B, can be considered as markers for sexual stages, since they are differentially expressed in the tick stages of the parasites, and not during its asexual blood stages (Alzan et al., 2016b). An important limitation for the study of the biology of sexual stages in *Babesia* parasites is their development in the midgut of the tick. The haemoglobin-rich milieu of the tick midgut is extremely complex due to the abundance of tick and bovine cells, which makes the identification of sexual forms of the parasite particularly difficult. A significant advance was the development of an in vitro system for the induction of sexual forms of *B. bovis* using blood cultures (Hussein et al., 2017) with similar systems developed for *B. bigemina* (Mosqueda et al., 2004, Bastos et al., 2013, Bohaliga et al., 2018). Use of this novel experimental tool will facilitate further clarification of the biology of the parasite, especially aspects related to its transition into sexual forms. In fact, this system already allowed confirmation of the expression of the 6-Cys A protein in the surface of the sexual stage forms using live immunofluorescence analysis (Hussein et al., 2017). Furthermore, the system was also essential for the characterisation of HAP2, a protein encoded by a highly conserved gene family also identified previously in *Plasmodium* (Angrisano et al., 2017). Malaria parasites, as well as other related apicomplexans, express a protein known as HAPLESS2/GCS1 (HAP2) on the surface of microgametes that occur in the mosquito gut lumen, which is also a candidate for TBV against *Plasmodium*. The *B. bovis* HAP2 was also found exclusively expressed in the surface of in vitro induced sexual forms, but not in in vitro cultured asexual stages of the parasite. Parasites with a KO in the HAP2 gene are able to grow in in vitro cultures, suggesting that the expression of this gene is irrelevant for its development in RBCs (Hussein et al., 2017). However, HAP2 KO *B. bovis* parasites cannot be induced into sexual stages using the in vitro induction system, suggesting that expression of HAP2 is required for the formation of sexual forms in *B. bovis* (Hussein et al., 2017). Altogether, this research suggested HAP2 protein as an additional prime candidate for the development of TBVs against *B. bovis*.

Additional novel proteomic tools were also recently applied to explore the proteome of kinete stages of *B. bovis*; a stage of the parasite that exclusively occurs during its development in ticks (Johnson et al., 2017). The study identified 10 proteins that are differentially expressed in kinetes that may also represent vulnerable targets for intervention against parasite transmission.

Finally, identification of proteins that are essential for the completion of the parasite life-cycle in the tick can also be used for the development of subunit vaccines that are not transmissible by ticks. Importantly, genes required in tick stages are usually irrelevant for the development of the parasites in asexual blood stages, greatly facilitating the process of KO parasites, since gene editing and transfection methods are usually applied in blood stage parasites and mutations affecting such non-essential genes generally result in viable blood stage parasites (Suarez et al., 2017). This can now be achieved by generating gene KO parasites, based on the use of recently developed *B. bovis* and *B. bigemina* gene manipulation methods (Silva et al., 2018a).

Overall, important progress has been made recently, leading to the identification of novel targets that can be exploited towards controlling bovine babesiosis. These advances occurred mainly by the application of novel KO techniques, in vitro sexual induction systems and proteomic techniques. However, it remains unknown whether TBVs based on the elicitation of antibodies that need to act in the milieu of the tick midgut, which has a distinct pH and is rich in proteases and other enzymes that can modify and inactivate the antibodies, is a workable approach. Future critical research is still required on testing new subunit vaccines based on tick stage antigens, with novel vaccine formulation and delivery systems that are able to elicit the high titers of antibodies likely required for the success of this novel vaccination approach. In addition, such subunit vaccine development requires considerable time to reach the end user, thus it would be feasible and practical to use attenuated parasite vaccines that cannot be transmissible in ticks. This can be possible, at least in theory, using KO approaches targeting genes required for the development of sexual stages of *Babesia* parasites.

### 3.2 Red blood cell invasion

*Babesia* sporozoites injected into the bovine host during tick feeding reach the circulation and invade RBCs (V#3, Table 2). RBCs are terminally-differentiated cells which lack any of their own genetic information and the ability to synthesise proteins, lipids and carbohydrates that, in addition to their low metabolic activity, makes for a relatively safe environment for pathogens to reside in. However, *Babesia*-infected RBCs are equipped with a source of introduced genetic information, which is now provided and operated by the parasite, and as a result, the parasite is relatively free to modify and control the red blood cell in order to fulfill its needs. Therefore, and similar to *Plasmodium* parasites, *B. bovis* dramatically modifies the composition and architecture of the infected RBCs (IRBCs). These changes include the protein and lipid composition of the RBC membrane (Florin-Christensen et al., 2000, Allred and Al-Khedery, 2006) and hence its relative permeability and deformability. Indeed, RBCs appear as ideal host cells for these parasite since they freely circulate through every organ of the host providing easy access to a variety of tissues that may be sites for capillary sequestration (in the case of *B. bovis*-IRBCs), they lack lysosomes and, importantly, are unable to present antigens to the immune system, thus facilitating escape of the parasite from immune effectors. RBCs are also unable to phagocytose and internalise other cells or nutrients by endocytosis, implying that the parasite must be the active force required for invasion to occur (V#3, Table 2). Merozoites reproduce asexually and mature inside RBCs until they egress and then invade new RBCs (V#4, Table 2). However, RBCs also transit through the spleen, an organ responsible for the clearance of IRBCs (V#5, Table 2).

*Babesia* parasites are equipped with an apical complex, a complement of secretory organelles that are required for RBC and target cell invasion. The apical complex organelle includes the pear shaped rhoptries, the microneme vesicles, spherical bodies (homologous to dense granules in *Plasmodium* and *Toxoplasma*) and other structures. The components of each of these organelles represent a knowledge gap in *Babesia*, but they are better defined in other related and more studied apicomplexans such as *Toxoplasma* and *Plasmodium* (Gubbels and Duraisingh, 2012). However, some apical complex molecules are species-specific and not widely conserved among apicomplexans, perhaps as a result of immune selective pressure during many years of co-evolution with distinct vertebrate hosts, and are poorly defined in *Babesia* parasites. However, *Babesia* parasites are equipped with a core of typical and highly conserved apicomplexan molecules with a known role in invasion such as AMA-1, TRAP, MIC-1, (Gaffar et al., 2004a, Gaffar et al., 2004b, Cerede et al., 2005) CLAMP (Suarez et al., 2017), and rhoptry neck proteins (Kemp et al., 2013), which are conserved among most apicomplexans.

In contrast to the related malaria parasites, *babesia* sporozoites solely infect RBCs, which is the only cell targeted for invasion in the bovine host (Mehlhorn and Shein, 1984, Chauvin et al., 2009). The process of RBC invasion by *Babesia* sporozoites and merozoites remains an important knowledge gap. Unraveling this process might lead to the design of novel methods for the control of bovine babesiosis. The mechanics of RBC invasion were revealed using transgenic parasites (Asada et al., 2012), but the molecular events involved remain obscure. Possibly, the initial event occurring in invasion is the recognition of a target cell by a gliding parasite apparently by random interactions, likely using glycosylphosphatidylinositol (GPI)-anchored surface exposed molecules such as MSA-1 and MSA-2 in *B. bovis* (Hines et al., 1992, Suarez et al., 2000, Florin-Christensen et al., 2002) and GP-45 *B. bigemina* proteins (Fisher et al., 2001), but it is also possible that it can be, at least in part, driven by recently discovered differences in the electric potential in the RBC and parasite surfaces (Scudiero et al., 2018). An important element involved in invasion is the high level of motility of the babesial invading sporozoites and merozoites, which is achieved by a gliding motion, provided by the activity of a highly efficient actin motor (Asada et al., 2012). Once the parasite is able to establish an interaction with a suitable RBC, it adheres then re-orients in order to have its apical end in direct contact with the surface of the RBC, resulting in high affinity docking involving membrane fusions, mediated by parasite microneme, and perhaps rhoptry, proteins. All these mechanisms, initial recognition, gliding, and re-orientation, can be potentially targeted by new control methods, either by drugs or vaccines. Intriguingly, similar to other apicomplexans, *Babesia* parasites form a parasitophorous vacuolar membrane (PVM) and reside within a vacuole immediately following invasion of the RBC but, in contrast to other related apicomplexans, the PVM is rapidly lost, leaving the parasite in direct contact with the cytoplasmic content of the RBC, as is consistently observed for *B. bovis*, *B. bigemina,* and *B. divergens* (Rudzinska et al., 1976, Potgieter and Els, 1976, Gohil et al., 2010). *Babesia* parasites contain genes encoding other widely conserved proteins such as serine rhomboid proteases which might be involved in releasing the parasite into the PV (Li et al., 2012), and so may also play important roles in the process of RBC invasion. The PVM is formed with the contribution of molecules secreted by the apical complex of the parasites, likely including the spherical bodies, which are homologous with the dense granules in *Plasmodium* and other apicomplexans in addition to the incorporation of host RBC proteins (Soldati et al., 2004, Repnik et al., 2015). Recent work has identified agents that are able to inhibit the egress of *Babesia* parasites (Pedroni et al., 2016), so future exploitation of these may not only directly facilitate the control of babesiosis, but importantly, also enhance our understanding of the mechanisms involved in the egress of the parasite from IRBCs.

### 3.3 Sequestration and persistence in infected animals

**Fig. 2:**
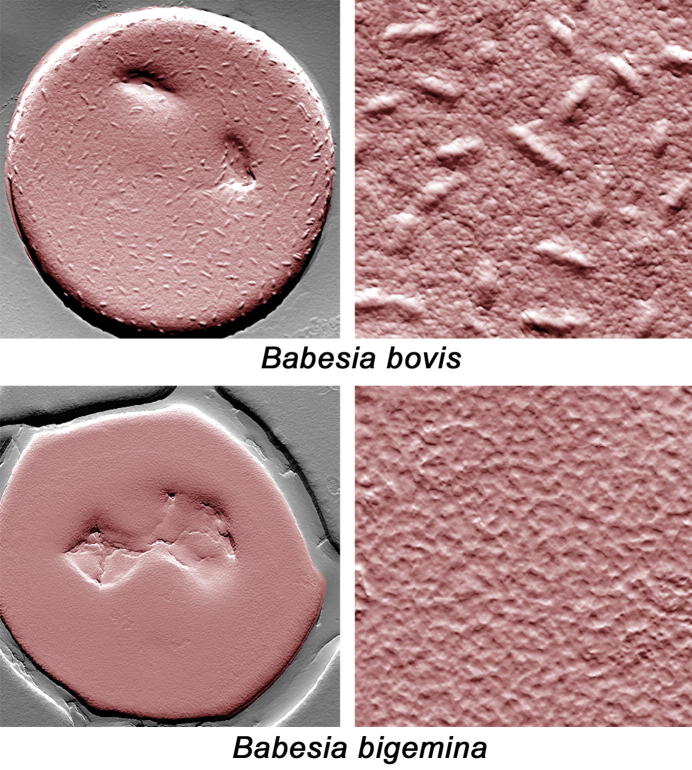
Pseudo-coloured atomic force microscopy images of the surface of bovine red blood cells (RBCs) infected with late stages of either *Babesia bovis* or *Babesia bigemina*. The unique ridge-like features present on the surface of *B. bovis*-infected RBCs are notably absent from RBCs infected with *B. bigemina*. The right-hand panels represent a higher magnification view of the surface of the infected RBCs shown in the left-hand panels. The atomic force microscopy appearance of the surface of normal, uninfected bovine RBCs is similar to RBCs infected with *B. bigemina* (not shown).

The growth of *Babesia* parasites in RBCs results in dramatic changes to the architecture of the IRBCs which display features that are highly atypical for normal RBCs (Gohil et al., 2010). These changes include modifications to the RBC that likely favor escape of the effector arms of the immune system by the parasite, such as increased cellular adhesiveness (in the case of *B. bovis*) and high antigenic variability. While *Babesia* parasites have a relatively small genome (approximately 7.0–16 Mbp) (Jackson et al., 2014), compared with the larger genome (∼23 Mbp) of related *Plasmodium* parasites (Gardner et al., 2002), it is efficiently used by the parasites to create their own defense mechanisms and modulate the host responses for long-term survival. Importantly, comparative genomic analysis of *Babesia* parasites demonstrated a large expansion of genes involved in antigenic variation, a mechanism used by the parasites for escaping the immune pressure of the vertebrate hosts. This is exemplified by the presence of the large *ves* (variable erythrocyte surface antigen) gene family comprising more than 150 genes in *B. bovis*. Interestingly, members of the *ves* family have also been identified in the genomes of both *B. bigemina* and *B. divergens* (Jackson et al., 2014). It is known that, at least for *B. bovis*, that the *ves* family of genes encodes proteins responsible for generating rapid antigenic variation, which allows the parasite to escape immune responses of the bovine host. Therefore, the presence of such large gene families suggests that periodic switching of variant antigens expressed on the surface of IRBCs is likely concomitant with the acquisition of new antibodies created by the vertebrate hosts, a phenomenon also observed in *P. falciparum* with the variant antigen determinants (*var*) gene family (Biggs et al., 1991, Allred et al., 1993). The consistent investment of *Babesia* parasites in developing a mechanism based on gene family expansion aimed at creating antigenic diversity is a strong indication of the importance for the need of such avoidance mechanisms in *Babesia* spp. At least for *B. bovis*, the mechanisms involved include the expression of variant genes in a “locus of active transcription” (LAT), but antigenic variation can also be potentiated, likely by the activity of segmental gene conversion mechanisms, that can provide increased sequence changes in these genes (Al-Khedery and Allred, 2006). In addition, RBCs infected with *B. bovis*, but not *B. divergens* or *B. bigemina*, can adhere to host vascular endothelial cells and accumulate in the microvasculature to avoid destruction in the spleen. In contrast, *B. bigemina* parasites can express IgM receptors on their surface (Echaide et al., 1998), although the mechanisms and consequences derived from the expression of such molecules in *B. bigemina* remain unexplained. Interestingly, similar IgM masking was also described to occur in malaria parasites as a mechanism to escape protective immunity (Barfod et al., 2011). These very intriguing findings are suggestive of the existence of an array of diverse evasion maneuvers that are employed by these parasites. Therefore, cytoadhesion and vascular sequestration in *B. bovis* appears to be mediated by VESA1 antigens, which are aptly expressed on the surface of the IRBCs. Similar to in the situation with malaria parasites, *B. bovis* affects changes to the surface architecture of IRBCs with the formation of ridge-like structures (Gohil et al., 2010) (Fig. 2). The molecular composition of these ridges has still not been identified but they likely contain the VESA1 antigens and are involved in the interactions of the IRBCs with vascular endothelial cells, resulting in cytoadhesion and their accumulation in the microvasculature. However, *B. bigemina* and *B. divergens*, despite expressing genes that are homologous to *ves* genes (Jackson et al., 2014), neither express ridges on the surface of IRBCs nor do IRBCs sequester in the microvasculature (Hutchings et al., 2007, Gohil et al., 2010, Scudiero et al., 2018). It is possible that such ridges on *B. bovis*-IRBCs act as a platform to anchor and cluster the cytoadhesion ligand on the IRBC (similar to knobs in *P. falciparum*) (Crabb et al., 1997, Gohil et al., 2010). The lack of such ridges on the surface of RBCs infected with either *B bigemina* or *B. divergens* may imply that the VES proteins are not correctly presented or anchored to be functional in vivo in these two non-sequestering parasites. However, the lack of cytoadhesion of *B. bigemina*-IRBCs may have a structural basis, since the VES proteins in *B. divergens* and *B. bigemina* lack cysteine-rich CKRD and VDCS domains (Jackson et al., 2014). In addition, *B. bigemina* and *B. divergens ves* genes seem not to be organised into a locus of active transcription (LAT) as described in *B. bovis* (Jackson et al., 2014). In addition, the Small Open Reading Frame (SmORF) family in *B. bovis* could assist in capillary sequestration together with VESA1 proteins in a manner similar to STEVOR proteins in *Plasmodium falciparum*. *Babesia bovis SmORF* genes are found within 4 kb of members of the *ves1* family; an arrangement that is similar to the location of *var*, *rifin* and *stevor* genes in *P. falciparum* (McRobert et al., 2004, Sanyal et al., 2012, Tibúrcio et al., 2012). Furthermore, the *SmORF* gene family is present only in *B. bovis* and not in *B. bigemina* or *B. divergens*, suggesting a potential role of the SmORF family in cytoadhesion. To fully understand the virulence mechanisms in bovine babesiosis caused by *B. bovis*, we also need to identify the bovine receptor for IRBCs expressed on the surface of microvascular endothelial cells. Based on studies of *P. falciparum*, CD36, also known to be expressed on the surface of bovine endothelial cells, is a possible candidate as a receptor for *B. bovis* IRBCs, but this still remains to be demonstrated. New experimental tools including transfection combined with cytoadhesion assays will be helpful to define the molecular mechanisms mediating the process of cytoadhesion and vascular sequestration of IRBCs. This could include testing mutated *ves* genes lacking the putative receptor region containing the cystein-rich domain, or the expression of *B. bovis* wild type and mutated VES proteins, or other functionally relevant RBC surface proteins in normally non-adhesive *B. bigemina* parasites. These experiments are now made possible using the recently developed stable transfection system for *B. bigemina* (Silva et al., 2018a).

**Fig. 3:**
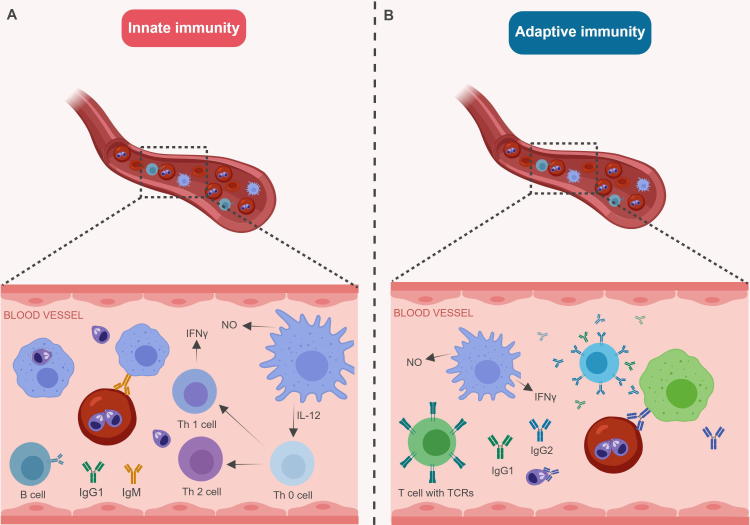
Schematic representation of protective immune responses in bovines infected with *Babesia* parasites. (A) Representation of innate immunity in young calves. The innate immunity in young calves is characterised by rapid activation of macrophages, abundant release of Interferon-γ (IFN-γ) and nitric oxide (NO). Young, naïve calves are naturally more resistant to infection and usually survive the challenge upon exposure to *Babesia*-infected ticks in endemic areas (a process also known as pre-munisation). In contrast, adult animals are more susceptible to *Babesia* infection and usually develop acute, often fatal, babesiosis. Animals which survive acute infections can develop chronic babesiosis and produce life-long protective immune responses. Further, innate immune responses appear to be more pronounced in young, rather than adult, animals. (B) Representation of adaptive immunity in persistently infected or vaccinated animals. Macrophages and protective neutralising antibodies appear to be essential for control of parasitemia in vaccinated and persistently infected animals. Figure generated using BioRender.

Until now, members of the VES family are the only *B. bovis* RBC surface-exposed proteins that have been characterised in any detail. However, the parasite is able to export additional, previously uncharacterised, proteins to the IRBC membrane and expose them on the cell surface to play important roles in host-parasite interactions (Gohil et al., 2013). This includes members of the poorly characterised SmORF gene family (Brayton et al., 2007, Ferreri et al., 2012). In fact, and similar to *Plasmodium* parasites, *B. bovis* has the ability to completely transform the architecture of the red blood cell in which it matures and divides. However, the proteins identified in *Plasmodium* parasites as responsible for similar changes seen in the architecture of the IRBCs are conspicuously absent in *Babesia* parasites, suggesting the operation of convergent evolutionary mechanisms resulting in the export of the required parasite proteins and in similar cytoadhesive phenotypes. A typical *B. bovis*-IRBC displaying surface modifications, as visualised by atomic force microscopy (AFM) is shown in Fig. 2. RBC modifications include the formation of RBC surface ridges that effectively increase the total surface area of the IRBC and appear to be essential to facilitate cytoadhesion of IRBCs in the host microvasculature, either by facilitating adhesion to endothelial cells or/and platelets (Hutchings et al., 2007). It is also possible that these modifications might also help the parasite avoid trapping by macrophages or interfere with the interactions with hosts’ antibodies, although all of these remain hypothetical (Scudiero et al., 2018). Consistently, no such RBC surface modifications are evident on the surface of non-cytoadhesive *B. bigemina*-IRBCs (Fig. 3) (Hutchings et al., 2007, Scudiero et al., 2018). It is likely that the changes in the IRBCs are mediated by proteins exported by the parasite, given that RBCs are terminally differentiated cells which lack any protein synthesis activity whatsoever, or alternatively, the result of the modification of existing RBC surface molecules by the parasite. Importantly, a *Plasmodium* export element (PEXEL)-like signal was identified in exported proteins in *B. bovis* such as Spherical Body Protein (SBP) 2. The bipartite functional domain ‘PEXEL’ appears to be an essential component of the molecular mechanisms involved in the export of *Plasmodium* proteins into the host cell. This domain consists of an N‐terminal eukaryotic signal sequence followed by a pentameric conserved motif with the consensus sequence, RxLxE/Q/D (Hiller et al., 2004, Marti et al., 2004). Exported proteins containing the PEXEL motif are directed to the endoplasmic reticulum, where they are subsequently cleaved by a protease (Boddey et al., 2010, Russo et al., 2010).

In addition, another form of antigenic variation in *Babesia* spp., based on allelic diversity in GPI-anchored variable merozoite surface antigens (VMSA) (Carcy et al., 2006), has also been identified at a population level (Suarez et al., 2000, Leroith et al., 2005, Berens et al., 2005, Lau et al., 2010). However, it remains premature to conclude that this mechanism could allow heterologous parasites to escape protective immunity as this could be due to variation in several genes and gene products that have not yet been explored. It is interesting that similar mechanisms for antigenic diversity and cytoadhesion co-evolved independently in distinct protozoa such as *Plasmodium* and *Babesia*. Antigenic variation using similar mechanisms have also been identified in other protozoans such as trypanosomes, and even prokaryotic organisms (Barbet et al., 2000) that can cause persistent disease. The presence of similar antigenic variation mechanisms that are useful to these otherwise distinct parasitic organisms which arose as a response to relatively similar and invariable immune systems of vertebrates is another interesting example of convergent evolution.

The concerted operation of these mechanisms results in parasites that are able to establish persistent infection of the bovine host, thus maximising their chances of transmission by tick vectors. Persistently infected bovines are apparently healthy and do not show clinical signs of *Babesia* spp. infection, unless the animal is exposed to stress, co-infections with other pathogens, or if it is immunocompromised, for example by splenectomy, which may result in the breakdown of its immunity to *Babesia* spp., and the resurgence of acute disease. It is not known exactly what factors are required for the bovine to remain apparently healthy during persistent infection. It is likely that by that point the immune response maturated enough to form high affinity antibodies and a robust cellular immunity based on regulatory CD4+ and regulatory CD8+ lymphocytes that secrete adequate classes and levels of cytokines which are able to keep in check the ability of the parasites to expand in the persistently infected animals (see below). Thus, it is possible that such a balance between a relatively healthy status and acute infection is provoked by constant immune stimulation by the parasite, an example of the establishment of a symbiotic relationship between the host and the parasite upon the development of persistent infection.

### 3.4 Immune responses to *Babesia* infection

The precise nature of protective immune responses involved in the control of acute babesiosis in bovines also remains an important research gap, largely due to practical, economic, and ethical restrictions associated with performing experiments in bovines and the lack of reliable small animal models for *B. bovis* and *B. bigemina*. Therefore, the lack of practical and reliable experimental systems for defining in detail the mechanisms involved in the development of protection critically affects our ability to design effective vaccines. Considerable progress was made in understanding the immune responses to *B. bovis* infections; however, the knowledge of immune responses to *B. bigemina* infections remains essentially unknown. Briefly, immunity to *Babesia* parasites in young animals and adult animals requires the strong induction of innate immune responses and development of effective adaptive immune responses, respectively (represented schematically in Fig. 3). It has also been suggested that successfully immunised animals and persistently infected animals that survive acute stage infections and are able to control parasitemia rely on antigen-specific CD4+ T cells that produce IFN-γ. This cytokine plays a central role since it can activate macrophages, is required for the clearance of parasites, and enhances production of the neutralising IgG2 antibody (Brown and Palmer, 1999, Homer et al., 2000, Brown, 2001, Estes and Brown, 2002). This antibody isotype, in combination with IgG1, was shown to passively protect cattle against homologous strain challenge (Mahoney, 1986). The identities of the antigens that are targeted by these protective neutralising antibodies, however, remain unknown. The mechanism of T cell activation and the role of the distinct T cell population (such as γδ-T cells) during a successful adaptive immune response in vaccinated/persistently infected adult cattle also remain to be explored. In summary, robust, innate immune responses are needed to survive the acute stages of infection, followed by efficient stimulation of immune mechanisms leading to the production of antibodies, which are critical effectors to control infection in vaccinated and persistently infected animals.

#### 3.4.1 Age-related immune responses to *Babesia* infections in cattle

Several reviews have described models of protective immune mechanisms for *B. bovis* (Goff et al., 1998, Brown and Palmer, 1999, Brown, 2001). Experimental evidence indicates that the resolution of acute *B. bovis* infection in immunologically-naïve young animals depends on a relatively strong innate immune response concomitant with infiltration of large leukocytes (monocytes/macrophages, Natural Killer (NK) cells and immature dendritic cells (DCs), macrophage activation by IFN-γ or parasite-derived products and priming of immune cells to release toxic macrophage metabolites including Nitric Oxide (NO) (Goff et al., 2010, Goff et al., 2003, Goff et al., 2001). A closer look at the immune responses in the spleen of young animals revealed the occurrence of cross-talk (mediated by IL-15 secreted by immature DCs) between the NK cells and immature DCs, resulting in IFN-γ secretion by NK cells and subsequent activation of DCs (Schneider et al., 2011). Further, efforts to understand the immunological basis for age-related resistance in the spleen of young (6-month old) and adult animals infected with *B. bovis* showed interesting differences in the cytokine response and NO release (Goff et al., 2001). Young animals showed increased expression of IL-12 and IFN-γ in spleen (3–6 days p.i.) 3 days earlier than adult animals and plasma levels of IFN-γ were also detected earlier in young animals than adult animals. Further, the sign of inducible nitric oxide synthase (iNOS) message was detected in young animals around day 7–8p.i., concomitant with parasite clearance. Many in vitro and ex vivo studies showed that IFN-γ secretion and NO release from immune cells were critical for parasite clearance, suggesting that the increased expression of IFN-γ and potential NO release are associated with increased resistance in young animals. As opposed to these observations, a few reports have shown that NO produced by macrophages during *B. bovis* infection was only partially growth inhibitory in in vitro experiments and in vivo experiments showed the limited role of NO in parasite clearance (Gale et al., 1998, Shoda et al., 2000). Interestingly, an earlier study showed that a soluble factor under 14 kDa isolated from calf serum and not from adult serum was strongly growth inhibitory to *B. bovis* replication, suggesting the presence of an alternative parasite growth inhibitory product in addition to NO in young animals (Levy et al., 1982). The soluble factor in the serum from young animals shown to have babesiacidal activity remains unknown to date. Therefore, the innate branch of the immune system plays a crucial role, mainly in the control of the expansion of the parasite in the early stages of infection, especially in naïve young animals. The efficient induction of these innate immune mechanisms in the young animals can lead to the development of a protective adaptive immune response in the adult animals and prevent the establishment of persistent disease, or the death of the infected animal due to the devastating effects of uncontrolled acute infection. Enhanced understanding of the mechanisms of resistance in young naïve animals to acute *B. bovis* infection (innate immunity) and those required for controlling parasitemia to persistent levels in adult cattle that survive infection (adaptive immunity), is also critically important for developing strategies to induce a protective immune response by vaccination, and more research is required in order to close this important research gap.

#### 3.4.2 Immunopathological effects on the host

The virulence of *Babesia* parasites is characterised by both parasite-mediated exploitation of the host and infection-mediated immunopathology. During an acute infection, especially in immunologically-naïve adult animals, *Babesia* parasites exploit the host and reproduce mostly unchecked, resulting in important destruction of host RBCs and a dramatic drop in haematocrit (Brown and Palmer, 1999). As a result, the infected host experiences decreased levels of oxygen in tissues and vital organs, causing apnea and respiratory distress, and responds to the infection with an increase in body temperature, with rectal fever that can go above 39 °C for several days. Hemolysis seems to be more dramatic in infections caused by *B. bigemina*, and the resulting hemolytic disease can be detected by the red color of the urine resulting from the secretion of molecules derived from the mechanisms of haemoglobin degradation in the liver. The high level of RBC destruction also causes jaundice and kidney damage, and the over-activity of the spleen resulting in part from IRBC trapping results in marked splenomegaly. It is possible that increased parasitemias usually found in *B. bigemina* infections, when compared with cytoadhesive and sequestering *B. bovis* parasites, also contribute to increased parasite trapping by the spleen concomitant with increased concentrations of haemoglobin and degradation products in the blood, resulting in increased kidney damage and haematuria in babesiosis caused by *B. bigemina*. In contrast, a hallmark of *B. bovis* infections is the presence of clinical neurological signs, concomitant with sequestration of parasite-IRBCs in the microvasculature of the brain. These two are the main and key signature pathological manifestations of acute bovine babesiosis in each of these parasites. Immunopathological effects on a naïve host are likely to be stronger due to the lack of immune system priming against *Babesia* infections and non-specific parasite clearance mechanisms (i.e. increased expression of inflammatory cytokines). The severe pathogenesis is thought to be partially immune-mediated, and over-production of soluble mediators including IFN-γ, TNF-α, and NO (Wright et al., 1988, Clark and Jacobson, 1998). Early induction (day 3–6p.i.) of TNF-α, IFN-γ, IL-12 and IL-18 together with late induction (≥day 10 6p.i.) of IL-10 by the immunocompetent cells of the host responding to the infection may result in increased inflammatory responses and damage in tissues containing sequestered IRBCs in *B. bovis* infections (Goff et al., 2002a, Goff et al., 2001, Goff et al., 2002b).

## 4 Current molecular toolbox and future perspectives

Despite numerous research gaps and biological challenges that must be overcome before we can generate more effective control methods to prevent or better control babesiosis, the constant influx of new discoveries and technologies in the field of both babesiosis and closely related apicomplexans, supports the successful development of new methods aimed at a more effective control of bovine babesiosis and, eventually, human babesiosis. Important advances in our understanding of the biology of the parasites can be directly and indirectly associated with the sequencing of relevant *Babesia* spp. genomes, which began with the publication of the first complete *B. bovis* genome in 2007 (Brayton et al., 2007). This was followed by the development of transfection systems for gene modification and functional analysis to accelerate vaccine candidate discovery (Suarez et al., 2017). Advancements in our understanding of the genomics of *Babesia* parasites has allowed, for instance, the complete characterisation of critical genes/gene families such as the large, antigenically-variable *ves1* gene family involved in host immune evasion, genes involved in the development of sexual stages (6-Cys, CCp, CPW-WPC, HAP2, etc.) (Bastos et al., 2013, Alzan et al., 2016b, Hussein et al., 2017) and conserved master regulatory genes such as AP2 (Alzan et al., 2016a). The AP2 and associated genes are responsible for transcriptional control of the genes involved in parasite stage transitions, as it was previously discovered in *Plasmodium* and *Theileria* parasites (Painter et al., 2011, Pieszko et al., 2015, Alzan et al., 2016a).

The subsequent application of other “omic” methods such as transcriptomics for example, permitted the comparison of virulent and attenuated strains of *Babesia* spp. in order to address the definition of virulence factors and attenuation markers, one of the most important remaining fundamental research gaps limiting rational vaccine design. The conclusion of genomic comparisons among virulent and attenuated paired strains was the finding of decreased population complexity in the attenuated strains compared with their virulent pairs, but the studies failed in identifying candidate genes or sets of genes responsible for increased or decreased virulence (Lau et al., 2011). Recent studies comparing transcriptomic profiles of geographically distinct virulent *B. bovis* isolates (Mexican, Australian and Argentinian strains) with their attenuated derivatives resulted in the identification of a limited number of differences such as the recent identification of the SBP2t11 gene as a marker for attenuation of *B. bovis* parasites (Pedroni et al., 2013, Gallego-Lopez et al., 2018). Other pioneering studies used transcriptomic and proteomic approaches to compare gene expression profiles of blood and kinete stages of *Babesia* spp. (Johnson et al., 2017) and showed that different sets of genes are activated and repressed in order to support the development of the parasite in the completely distinct environments of the host and the tick. While the functions of the genes identified in this work remain unknown, this can also be addressed in the future using transfection, KO and gene-editing approaches.

Critical to future research is a complete elucidation of the molecular mechanisms involved in the establishment of persistent disease by these parasites. This includes the dissection of the mechanisms of persistence at a molecular level. We must also improve our mechanistic understanding of the ability of *B. bovis*-IRBCs to cytoadhere and sequester, include the identification of the changes in the architecture of the surface of the IRBCs and the mechanisms involved in the expression of *ves* genes in this parasite. Additionally, nothing is known about the role of these VES proteins in *B. bigemina* and the mechanisms used by this parasite for persistence. Discovering these mechanisms will guide new strategies and identify potential targets toward rational subunit vaccine design. More widespread use of currently available tools such as in vitro static and dynamic (flow-based) cytoadhesion assays (Hutchings et al., 2007, Gohil et al., 2010), that can model parasite sequestration in vivo, would provide better insight into these mechanisms and help with the identification of new therapeutic agents that inhibit cytoadhesion. Increased understanding of the factors involved in induction of strong innate immunity in young animals will provide educated ideas/strategies for adjuvant selection and vaccine design. Understanding the mechanism of strong innate immunity in young animals will inform the type of vaccine-induced response needed to control parasitemia. In addition, careful consideration must be given to adjuvant selection and vaccine design as over-production of soluble mediators of cell-mediated immune responses might lead to increased pathology in vaccinated animals. A close evaluation of protective immune mechanisms involved in the efficient clearance of IRBCs by the spleen of young animals and understanding the balance of protective type 1 immune responses is important to design an effective subunit vaccine with limited vaccine-associated pathology in animals.

Recent work has focused on development of in vitro sexual-stage induction systems, due in part to the practical difficulties associated with the study of tick stages in the tick midgut milieu. Application of this system to *B. bovis* has identified the requirement of the HAP2 gene for the development of sexual-stage parasites and its link with the expression of the sexual-stage marker genes 6-Cys A and B (Hussein et al., 2017). The recent development of this in vitro method for the induction of *Babesia* sexual stages from cultured parasites will facilitate the study of the antigenic and morphological changes that the parasites undergo, the definition of possible cell surface-exposed immunological targets, and the testing of the effects of specific antibodies against key antigens (Camacho-Nuez et al., 2017, Hussein et al., 2017, Bohaliga et al., 2018). These developments can potentially accelerate the rate of discovery towards the development of transmission blocking vaccines.

Taken together, these new data and newly developed genetic manipulation systems are fundamental for the development of novel, next-generation vaccines that can target multiple stages of the parasites’ life-cycle, which may be essential for the eventual control of this disease.

## 5 Conclusions

Advancement of research in the biology of *Babesia* parasites is heavily influenced by the constant advances in molecular and cellular biology, immunology, computational sciences and vaccinology. In this review, we have attempted to link the need for a more complete understanding of the biology of *Babesia* parasites, including a better and more detailed characterisation of different phases in the parasite life-cycle and the distinct phases of the interactions of *Babesia* parasites with their host. Overall, the road ahead is still long and winding. However, we have also presented evidence supporting that we are acquiring an appropriately-equipped toolbox for guiding us on a successful journey towards babesiosis control. In practical terms, research will require improved and reliable experimental systems, especially when the natural systems present difficult practical obstacles and ethical concerns.

Currently, robust transfection methods are available for genetic modification in *B. bovis* and *B. bigemina*. In addition, our ability to efficiently manipulate the *Babesia* genomes will be augmented by the development of CRISPR/Cas9 gene editing methods. These gene manipulation methods combined with in vitro static and haemodynamic assays for the analysis of the interactions between IRBCs and host vascular endothelial cells, as well as studies using transgenic parasites in vivo, will allow a more detailed dissection of cytoadhesive mechanisms leading to the creation of new vaccines that might interfere with parasite adhesion and sequestration. The future development of efficient sub-unit vaccines against bovine babesiosis remains a challenge. Importantly, more research is needed to better understand the nature of protective immune responses in young animals and successfully vaccinated animals, and inform effective strategies for vaccine design. Additionally, other current and future methods of analysis, such as in vitro tick artificial feeding systems (Trentelman et al., 2017), will also facilitate the study of the interactions of the parasites with their tick hosts. These techniques, added to the in vitro sexual-stage induction methods, will provide a detailed analysis of the parasite-tick interface, which will allow in turn the identification and simplified method of testing of vaccine candidates with the potential to block the transmission of the parasite. Evaluation of the most promising antigens, those with the potential to limit blood stage infection and tick transmission, as vaccines in cattle in proof of concept trials will aid in the selection of handful of antigens with effective protection efficacy. Ideally, a multivalent subunit vaccine containing the selected antigens would be designed with the purpose of blocking both acute infection and transmission of the parasites.

Finally, although we may gain understanding of the function of individual genes and their biological relevance in different life-cycle stages of the parasite, a better understanding of the ‘big picture’ of this disease is still needed. Clearly, controlling the parasite presents an immense, but important, intellectual challenge, but creative and effective use of the current and future technical resources suggest that the ‘sky is our limit’ in our search for improved control measures for babesiosis that impose an important burden for humans and for food production, with an ultimate impediment to the progress of humanity in the future.
